# The role of chalcogen vacancies for atomic defect emission in MoS_2_

**DOI:** 10.1038/s41467-021-24102-y

**Published:** 2021-06-22

**Authors:** Elmar Mitterreiter, Bruno Schuler, Ana Micevic, Daniel Hernangómez-Pérez, Katja Barthelmi, Katherine A. Cochrane, Jonas Kiemle, Florian Sigger, Julian Klein, Edward Wong, Edward S. Barnard, Kenji Watanabe, Takashi Taniguchi, Michael Lorke, Frank Jahnke, Johnathan J. Finley, Adam M. Schwartzberg, Diana Y. Qiu, Sivan Refaely-Abramson, Alexander W. Holleitner, Alexander Weber-Bargioni, Christoph Kastl

**Affiliations:** 1 Walter Schottky Institut and Physics Department, Technical University of Munich, Garching, Germany; 2 Munich Center for Quantum Science and Technology (MCQST), München, Germany; 3 Molecular Foundry, Lawrence Berkeley National Laboratory, Berkeley, CA USA; 4 nanotech@surfaces Laboratory, Empa – Swiss Federal Laboratories for Materials Science and Technology, Dübendorf, Switzerland; 5 Department of Molecular Chemistry and Materials Science, Weizmann Institute of Science, Rehovot, Israel; 6 Department of Materials Science and Engineering, Massachusetts Institute of Technology, Cambridge, MA USA; 7 Research Center for Functional Materials, National Institute for Materials Science, Tsukuba, Japan; 8 International Center for Materials Nanoarchitectonics, National Institute for Materials Science, Tsukuba, Japan; 9 Bremen Center for Computational Materials Science, University of Bremen, Bremen, Germany; 10 Bremen Institute for Theoretical Physics, University of Bremen, Bremen, Germany; 11 Department of Mechanical Engineering and Materials Science, Yale University, New Haven, CT USA

**Keywords:** Two-dimensional materials, Electronic properties and materials, Two-dimensional materials

## Abstract

For two-dimensional (2D) layered semiconductors, control over atomic defects and understanding of their electronic and optical functionality represent major challenges towards developing a mature semiconductor technology using such materials. Here, we correlate generation, optical spectroscopy, atomic resolution imaging, and ab initio theory of chalcogen vacancies in monolayer MoS_2_. Chalcogen vacancies are selectively generated by in-vacuo annealing, but also focused ion beam exposure. The defect generation rate, atomic imaging and the optical signatures support this claim. We discriminate the narrow linewidth photoluminescence signatures of vacancies, resulting predominantly from localized defect orbitals, from broad luminescence features in the same spectral range, resulting from adsorbates. Vacancies can be patterned with a precision below 10 nm by ion beams, show single photon emission, and open the possibility for advanced defect engineering of 2D semiconductors at the ultimate scale.

## Abstract (web summary)

The relation between the microscopic structure and the optical properties of atomic defects in 2D semiconductors is still debated. Here, the authors correlate different fabrication processes, optical spectroscopy and electron microscopy to identify the optical signatures of chalcogen vacancies in monolayer MoS2.

## Introduction

Control over atomic defects is the foundation of today’s semiconductor technology. For two-dimensional van der Waals semiconductors, the term “defect engineering” has been coined to suggest that, by introducing defects, these materials can be engineered beyond the established concepts of doping or alloying^1^, enabling advanced functionality, such as single photon sources^2,3^ or photocatalysis with chemical specificity^1^. Nevertheless, the microscopic understanding of defect-related modifications remains elusive due to a lack of thorough correlation between atomic structure and resulting macroscopic electronic and optical properties. Combing controlled defect engineering with optical spectroscopy as well as atomic imaging and ab initio theory, we identify the optical signature of pristine chalcogen vacancies in MoS_2_. Vacancies introduce a deep center with sharp optical emission, markedly different from previously observed broad luminescence bands^4–8^. Comparing annealed *vs*. He-ion treated MoS_2_, we establish that the recently discovered single-photon emitters in He-ion irradiated MoS_2_ originate from chalcogen vacancies^3^. The latter can be deterministically created with a precision below 10 nm^9^, underscoring the potential of defect engineering for two-dimensional (quantum-) optoelectronics.

In semiconductors, the interaction of free excitons with the Coulomb potential of lattice defects results in localized defect-exciton complexes^10^. In the traditional picture, exciton localization at shallow defects introduces an additional binding energy. Therefore, optical signatures of defects lie energetically below the free exciton^10^. Defect levels deep inside the band gap provide further relaxation pathways at even lower transition energies^10^. Since in two-dimensional (2D) semiconducting transition metal dichalcogenides, including MoS_2_, MoSe_2_, WS_2_, and WSe_2_, the screening of the defect potential is weak, and also the exciton Bohr radius is small (~ 2–3 nm)^11^, excitons are strongly confined in real space when coupling to defects^12^. Therefore, as shown theoretically for MoSe_2_ and MoS_2_, chalcogen vacancy levels give rise to a series of bound defect excitons, which then hybridize with excitonic states of the pristine system^12,13^.

At low-temperatures, most 2D semiconductors exhibit broad sub-gap emission extending several hundred meV below the exciton^4,5,7,8,14^. Generally, this sub-gap luminescence becomes stronger with increasing number of point defects present in the TMD layer, and it is often called L-band (emphasizing localization) or D-band (emphasizing defects) in the literature. The correlation between defects and the L-band was found either by introducing additional defects^5,14^, or by correlating spatial fluctuations of the existing defect density and excitonic properties^4,15,16^. Nevertheless, there is a surprising lack of consensus about the origin for such broad defect emission as well as about the detailed impact of specific defects on the excitonic properties. For example, some studies report a brightening of the room temperature exciton emission with increased defect density^6,17^, while other studies report an anticorrelation between defect density and PL yield^16,18^. For the low-temperature L-band, some studies emphasized radiative recombination at intrinsic point defects as underlying mechanism^4,7^. Other studies highlighted the relevance of molecular adsorbates. For example, calculations suggest that adsorbed molecular oxygen modifies the electronic structure of the sulfur vacancy in MoS_2_, either by removing the in-gap state^19^ or by *p*-doping via hole transfer from the absorbed molecular oxygen^6^. In this context, a brightening of room temperature excitonic emission in MoS_2_ upon oxygen plasma annealing was attributed to p-type counter-doping caused by chemisorption of oxygen radicals at sulfur defect sites, but no systematic correlation to defect emission was established^6^. Furthermore, it has been suggested that laser illumination incorporates atomic oxygen into pre-existing chalcogen vacancies either by photo-assisted dissociation of molecular oxygen^20^ or water^21^. However, chalcogen vacancies are likely already passivated by atomic oxygen in as-prepared TMDs^9,22,23^. Moreover, several studies demonstrated that laser annealing in controlled gas environments^24,25^ or encapsulation in hBN^26^ can completely remove the L-band suggesting chemisorbed or physisorbed molecules as its origin^25^.

By contrast, a spectrally sharp sub-gap luminescence and single photon emission was also reported at low-temperatures^2^. As the microscopic model, a combination of strain potentials, which funnel and localize excitons, and atomic defects, which provide recombination centers for localized excitons, was suggested^2^. While these point-like emitters appear randomly in as-prepared samples, they can be generated deterministically via engineered nanoscale strain potentials^2^. Recently, our group has demonstrated that atomic point defects, which are created deterministically by focused He-ion irradiation using a helium ion microscope (HIM)^9^, act as narrow and reproducible single-photon emitters, yet without a local strain potential^3,27^. Here, we disentangle broad defect emission due to adsorbates, which can be desorbed by in-vacuo annealing at moderate temperatures, and narrow defect emission via sulfur vacancies, which are generated both by annealing at high temperatures and He-ion irradiation. Combing controlled defect engineering with optical spectroscopy as well as atomic imaging and ab initio theory, we identify the optical signature of chalcogen vacancies in MoS_2_.

## Results

### Defect luminescence in MoS_2_

**Fig. 1:**
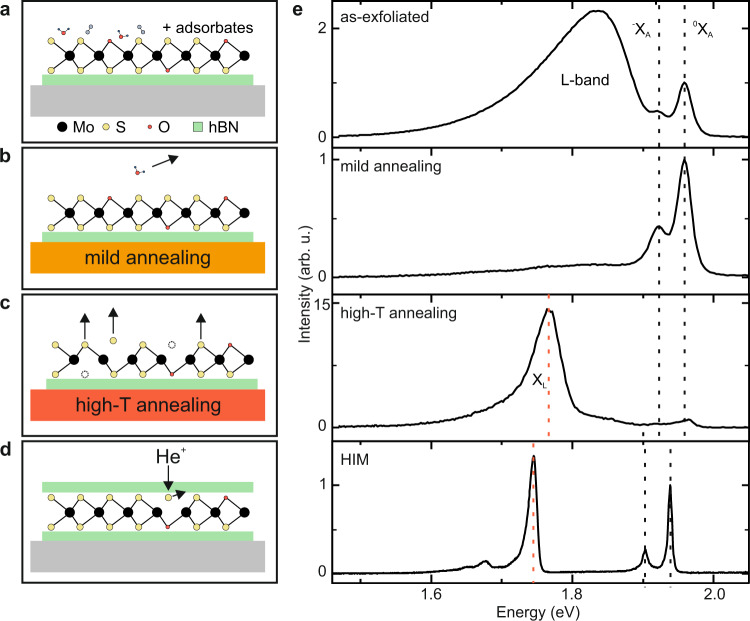
Optical emission induced by adsorbates and engineered point defects in single-layer MoS_2_. **a** Schematic of as-exfoliated single layer MoS_2_ on hBN (green) supported on a Si/SiO_2_ substrate (gray). Black (yellow) dots denote molybdenum (sulfur) atoms. Red dots denote oxygen atoms, which are present either as part of adsorbates or as substitutional atoms on sulfur sites. **b** Heating the substrate to mild annealing temperatures (*T*_annealing_ < 500 K, orange) removes the absorbates. **c** Heating the substrates to high annealing temperatures (*T*_annealing_ > 500 K, red) creates sulfur vacancies by thermal desorption (arrows). **d** Schematic of vacancy generation in MoS_2_ fully encapsulated in hBN through He-ion (He^+^) bombardment. **e** Evolution of low-temperature (*T* ∼20 K) photoluminescence spectra. As-exfoliated MoS_2_ exhibits a broad sub-gap luminescence (L-band) due to adsorbates. Mild annealing at *T*_annealing_ =  500 K removes these adsorbates suppressing the L-band. Upon annealing at T_annealing_ =  800 K, a narrow defect luminescence at 1.75 eV emerges (X_L_), due to the thermal generation of sulfur vacancies with well-defined in-gap states. Vertical, dashed lines indicate the emission energy of the neutral exciton (^0^X_A_), the trion (^−^X_A_), and X_L_. The intensities are normalized to the exciton transition ^0^X_A_. Helium-ion (He^+^) irradiation of MoS_2_ encapsulated in hBN using a helium ion microscope (HIM) generates a similar defect feature at 1.75 eV (*T* = 4.2 K).

Figure 1a–d show schematic atomistic representations of MoS_2_ at different stages of the annealing process. The corresponding low-temperature photoluminescence (PL) spectra of monolayer MoS_2_ on hexagonal boron nitride (hBN) are depicted in Fig. 1e. In addition to exciton (^0^X_A_) and trion (^−^X_A_)^11^, as-exfoliated MoS_2_ exhibits a prominent L-band from approximately 1.5–1.9 eV. Mild annealing (*T*_annealing_ = 500 K) in vacuum results in a striking reduction of the L-band, presumably due to desorption of adsorbates. Further annealing at high temperature (*T*_annealing_ = 800 K) introduces a narrow peak X_L_ at approximately 1.75 eV. A similar, yet even sharper, spectral signature is observed in fully encapsulated MoS_2_ after He-ion irradiation. We note that the data on the ion treated sample was measured on fully encapsulated MoS_2_ with top and bottom hBN, which is necessary for the ex-situ He-ion bombardment, whereas the data on the thermally annealed sample was measured on half-encapsulated MoS_2_, with bottom hBN only. The small exciton red-shift in the He-ion treated sample can likely be attributed to the increased dielectric screening in the fully encapsulated samples^28^. Additionally, inhomogeneous broadening effects due to a locally varying dielectric environment near the defects may result in an uncertainty of the defect emission line on the order of several meV^29,30^. The improved inhomogeneous broadening agrees with previous studies of fully hBN encapsulated heterostructures^26^. In the following, we show that adsorbates introduce a continuum of defect states, which is responsible for the L-band emission, whereas pristine sulfur vacancies, i.e., sulfur vacancies not passivated by atomic oxygen or decorated by molecular species, introduce a deep center, which is very likely also the origin of recently discovered single photon emission in He-ion treated MoS_2_^3,27^.

### Defect luminescence induced by thermal annealing

**Fig. 2:**
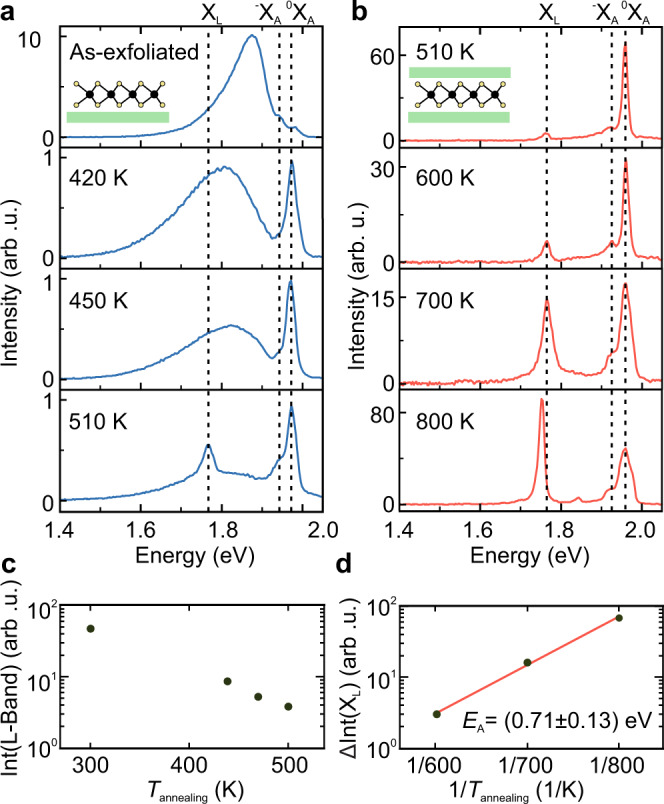
Evolution of MoS_2_ low-temperature PL with increasing annealing temperature. **a** Compared to as-exfoliated MoS_2_ on hBN (top panel) the broad L-band is strongly reduced after successive mild annealing. At 510 K, an emission line X_L_ appears at 1.75 eV (bottom panel). The spectra were normalized to the intensity of the exciton to highlight relative changes between the L-band, the trion and the free exciton emission. **b** PL spectra of fully encapsulated MoS_2_. With increasing annealing temperature, the intensity of X_L_ increases. Dashed lines highlight neutral exciton (^0^X_A_), trion (^−^X_A_), and X_L_. The spectra are presented as measured without further normalization. **c** Integrated intensity of the L-band in **a** as function of annealing temperature. The change in L-band emission is related to the desorption rate of adsorbates from the MoS_2_. **d** Arrhenius plot of thermal defect generation rate extracted from the change in intensity of X_L_ between subsequent annealing steps, which is denoted as ΔInt(X_L_). The activation barrier *E*_A_ extracted from the fit (red line) is (0.71 ± 0.13) eV consistent with formation of sulfur vacancies.

Figure 2 shows low-temperature PL of MoS_2_ after stepwise in-vacuo annealing up to 800 K. In each cycle, the samples were rapidly annealed in a customized cryostat for 30 min, and then cooled back to cryogenic temperature (*T*_sample_ ∼20 K) for PL characterization maintaining a high vacuum (*p* < 10^−4^ mbar) at all times (Supplementary Note 1). Figure 2a illustrates spectra of as-exfoliated MoS_2_ on hBN and after several mild annealing steps to 420, 450, and 510 K. Again, the as-exfoliated flake exhibits a prominent L-band (cf. Fig. 1a). The intensity of the L-band decreases relative to the intensity of the free exciton emission by one order of magnitude after annealing to *T*_annealing_ = 420 K, and it gradually disappears for higher annealing temperatures (Fig. 2b). Furthermore, the trion emission decreases initially compared to the free exciton indicating a reduced doping, as observed previously in TMDs during desorption of physisorbed gas^31^ and chemical dopants^32^. Hence, we attribute the L-band to adsorbates, which are progressively removed during the mild annealing steps. The desorption does not follow a simple Arrhenius law, since it depends on the total number of adsorbates, which is unknown. Therefore, we can estimate only an upper bound of ~100 meV for the desorption barrier, which agrees with ab initio studies for molecular adsorbates on MoS_2_^33^ and temperature programmed desorption on bulk MoS_2_^34^.

At *T*_annealing_ = 510 K, a spectrally narrow emission line X_L_ appears around 1.75 eV. In contrast to the L-band, the intensity of X_L_ increases with higher annealing temperatures, until the whole PL signal disappears at *T*_annealing_ > 700 K (Supplementary Note 2). To extract the thermal activation barrier of X_L_, we use an hBN/MoS_2_/hBN heterostack, where the L-band is already suppressed in the as-prepared structures. As seen in Fig. 2c, X_L_ brightens in the encapsulated monolayer with increasing annealing temperature, and further narrows after annealing to 800 K indicating a complete removal of residual adsorbates. At even higher temperatures (*T*_annealing_ = 900 K), the intensity of X_L_ decreases drastically, followed by the complete disappearance of the overall PL (Supplementary Note 2). Consistent with saturating a finite density of localized defect levels, X_L_ exhibits a saturating behavior as function of excitation power (Supplementary Note 3)^27^. In a simple rate equation model, the saturated defect emission is then proportional to the total number of emission centers. In this case, we can readily determine the energy barriers for defect generation since it is proportional to the difference in the integrated spectral weight of X_L_ compared to the previous annealing step. For example, the number of defects generated during annealing at 600 K is proportional to the difference in integrated PL intensity, which we label ΔInt(X_L_), after annealing to 600 and 510 K. In the limit of low density, defect generation is independent of the number of existing defects, and we expect a simple Arrhenius law. From Fig. 2d, we find an activation energy of (0.71 ± 0.13) eV for the X_L_-peak consistent with theoretical predictions for the formation energy of mono-sulfur vacancies (approximately 1 eV)^35,36^. The error was calculated from the standard deviation of the fit parameter for the slope. Interstitial sulfur defects should not form under high-vacuum, i.e., sulfur-poor, conditions^35^. The formation energies for transition metal vacancies are much larger (3–8 eV)^35,36^. Consequently, the formation of sulfur vacancies during annealing is thermodynamically the most favorable and, therefore, most likely process. To independently corroborate the value of the thermal activation energy and to quantify the absolute number of defects generated at a given temperature, we conducted a Raman study of additional, thermally annealed samples (Supplementary Note 4). At large enough defect densities, the inter-defect distance, or equivalently the absolute value of the defect density, can be inferred from a shift of the characteristic Raman modes due to phonon confinement effects^30,37,38^. From these experiments we extract an activation energy of (0.48 ± 0.23) eV for thermal defect generation, which agrees well with our in-situ study within the experimental uncertainty. For high annealing temperatures (*T*_annealing_ = 900 K), we can even determine the absolute value of the reaction rate to be on the order of 5 × 10^13^ vacancies per cm² per hour. However, we note that this value should only serve as a rough order of magnitude estimate.

### Atomic-scale identification of sulfur vacancy defects

We continue to corroborate the dominant generation of sulfur vacancies during annealing by atomic-scale characterization. Here, we perform high-resolution low-temperature scanning tunneling microscopy (STM) and atomic force microscopy (AFM) of single-layer MoS_2_ before and after high-temperature annealing in vacuum (*T*_annealing_ > 500 K) as well as before and after He-ion irradiation. These experiments are conducted on graphene/SiC heterostructures. The graphene substrate is essential as conductive support, but it quenches the defect emission, which is slow (100 ns–1 µs)^3,29,30^, and therefore the fast exciton emission (~10 ps) dominates on graphene (Supplementary Note 5)^16,39^. For STM, all samples were prepared in-vacuo by a mild annealing step in vacuum (*T*_annealing_ < 500 K) to remove adsorbates. Several STM studies established correlations between defect densities and (opto)electronic material properties of transition metal dichalcogenides, via large scale imaging at moderate resolution and counting of localized features^18,40,41^. In the following, we focus on the exact identification of atomic defects via high-resolution imaging of the orbital structure as well as independent corroboration of the lattice site, via AFM and ab initio simulations^22^. In agreement with our optical studies, the surface of MoS_2_ is virtually free from adsorbates after mild annealing. By far the most dominant defects in pristine material were oxygen atoms substituting sulfur, but no sulfur vacancies were observed within our statistics (Supplementary Note 6)^9,22,23^. The latter observation is in line with previous studies on MoSe_2_ grown by molecular beam epitaxy and WS_2_ grown by chemical vapor deposition, where in as-grown samples the dangling bonds of sulfur vacancies are effectively passivated by chemically bound atomic oxygen in the trigonal lattice configuration of the transition metal dichalcogenide^22,23^.

**Fig. 3:**
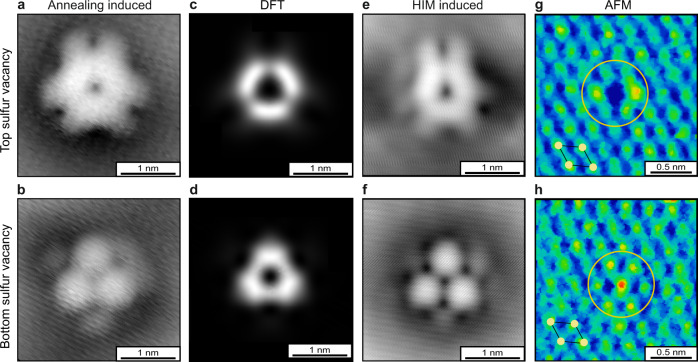
Scanning probe microscopy of sulfur vacancies in single-layer MoS_2_ on graphene. **a**, **b** Scanning tunneling microscope (STM) images of annealing induced vacancies in the top and bottom sulfur layer. The STM imaging parameters were tip bias *V*_bias_ = 450 mV, tunneling current *I*_t_ = 100 pA. **c**, **d** Density functional theory (DFT) calculations of 7 × 7 supercell containing one sulfur vacancy. The image displays a constant height slice 4.5 Å above and below the MoS_2_ layer corresponding to the charge density from sulfur vacancies in the top and bottom sulfur layer, respectively. **e**, **f** STM images of He-ion (HIM) induced top and bottom sulfur vacancies. **g**, **h** Atomic force microscopy (AFM) images of the top and bottom sulfur vacancies. The centers of the circles highlight the position of the vacancy defect. The pictogram indicates the sulfur lattice (yellow dots).

After additional high-temperature annealing, we observed only two additional types of defects within our experimental statistics (Fig. 3). Their high-resolution STM topography exhibits a trigonal symmetry (Fig. 3a, b), consistent with a vacancy either in the top or bottom sulfur lattice. For both vacancies, the charge densities calculated by DFT (Supplementary Note 7) at a distance of 4.5 Å above the MoS_2_ layer (similar to experimental conditions in STM) are shown in Fig. 3c, d. The calculations are in good agreement with the STM topography. The top vacancy appears trigonally ring-shaped, whereas the bottom vacancy appears triangular-shaped with bright maxima at the corners. These same defects, i.e., top and bottom sulfur vacancies, were also confirmed in the He-ion irradiated samples (Fig. 3e,f). For He-ion treated samples, sulfur vacancies are the dominant defect type, among other defects that are generated with lower yield^9^. Furthermore, we performed CO-tip AFM on the sulfur vacancies (Fig. 3g,h). For the top vacancy, we observe an apparent depression at the sulfur site, whereas for the bottom vacancy structural relaxation results in a slight protrusion, in agreement with previous studies on WS_2_^23^. Most importantly, AFM conclusively assigns the defect onto the sulfur sublattice, which is difficult from STM alone^20^. Overall, the scanning probe experiments confirm the composition and surface condition of MoS_2_ derived from optical characterization (cf. Fig. 1). From our different annealing experiments, we conclude that defect luminescence X_L_ at 1.75 eV arises from pristine, i.e., undecorated, sulfur vacancies. We note that, there are at least two pathways for formation of sulfur vacancies, which are desorption of a sulfur atom or desorption of an oxygen atom from a sulfur site. The latter substitute for sulfur in as-prepared TMDs^22,23^. Based on the similarity of the optical spectra and the abundance of sulfur vacancies in thermally annealed as well as in He-ion irradiated MoS_2_ (cf. Fig. 1), we propose that also the origin of quantum emission from individual He-ion induced defects is due to the (non-passivated) sulfur vacancy^27^.

### Excitonic defect states from ab initio theory

**Fig. 4:**
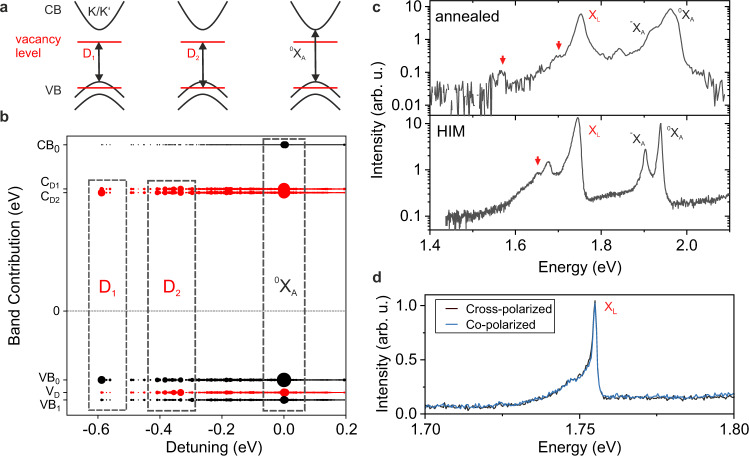
Defect bound excitons in monolayer MoS_2_. **a** Quasiparticle conduction (CB) and valence (VB) bands (black) near K-points and K′-points in the Brillouin zone of single-layer MoS_2_. The red lines denote states arising from sulfur vacancy levels. Arrows highlight the three types of excitonic transitions: between the delocalized bands (^0^X_A_), between the localized defect states only (D_2_) and between the valence band and the localized defect state (D_1_). **b** Band contribution to exciton transitions computed with GW-BSE. Dot size represents the relative oscillator strength, and the probability amplitudes of the occupied and empty bands contributing to it. Black indicates delocalized band states and red indicates localized defect states. CB_0_ and VB_0_, VB_1_ denote the dispersive unoccupied conduction and occupied valence bands (above and below the Fermi level), respectively. C_D1_ and C_D2_ are unoccupied localized defect levels, split by spin–orbit interaction arising from conduction band states. *V*_D_ is the occupied localized defect level arising from the valence band. The three main peaks regions assigned with the D_1,_ D_2_, and ^0^X_A_ transitions are marked with dashed lines. **c** PL spectra of annealed and HIM-treated MoS_2_. The defect emissions occur in a range of 0.2–0.3 eV below X_A_ with a dominant emission line (X_L_) around 1.75 eV. Further features (red arrows) are resolved at even larger detuning from the free exciton, in qualitative agreement with the predicted continuum of excitonic defect states below the pristine optical gap. **d** A single He-ion induced emitter shows no detectable valley polarization (*T* =  10 K, excitation energy 2.1 eV) as expected for transitions involving only defect levels (D_2_).

In a simple single-particle picture, three types of excitonic transitions can be qualitatively distinguished (Fig. 4a), similar to the approaches in ref. ^12^ for MoSe_2_ and ref. ^42^ for WSe_2_. However, taking many-body effects into account, the strong electron–hole interaction leads to a vast manifold of excitonic transitions for the defective crystal with varying eigenenergies and mixed degrees of pristine and defect-like character, which all contribute to the excitonic spectrum^12,13^. Figure 4b shows the excitonic spectrum and its band contributions from calculations of defective MoS_2_ (5 × 5 supercell corresponding to 2% of vacancies) within the GW-Bethe–Salpeter equation (GW-BSE) approach^43,44^. Each set of dots at a given energy describes an excitonic eigenstate of the defective MoS_2_ crystal. The size of the dots is proportional to the contributions from different bands, integrated across the Brillouin zone, to a particular excitonic state. This detailed evaluation of the band contributions suggests that transitions at the optical gap (^0^X_A_) have a substantial component from unbound electronic states between valence and conduction bands at K and K′ valleys (see also Supplementary Note 8). The next series of transitions (D_2_) occur predominantly between the resonant defect state overlapping with the valence band and the localized in-gap states. In the dilute limit, the localized defect states are k-independent, such that defect–defect transitions cannot exhibit valley-selectivity^12^. The lowest series of transitions (D_1_) couples the localized in-gap state to dispersive states in the valence band near K and K′, and they are predicted to show valley selectivity and corresponding polarization.

In Fig. 4c, we show experimental PL emission spectra of MoS_2_ with vacancies, introduced in encapsulated MoS_2_ both by in-vacuo thermal annealing and by ex-situ helium ion modification. Notably, while the dominant defect emission (X_L_) occurs about 0.2 eV below ^0^X_A_, we consistently observe weak emission features (red arrows) at even lower energies^3^. The multiple sub-gap emission peaks are qualitatively in agreement with the manifold of exciton states predicted by the calculations of the defective MoS_2_ (Fig. 4b). The calculated absolute energy of the features associated with transitions of type D_2_ is centered at 1.8 eV, which is consistent with defect emission at 1.75 eV, within the overall computational uncertainty on the order of 0.2 eV (Supplementary Note 9). Figure 4c further corroborates this assignment. Here, we plot the emission spectrum of a single defect, which was generated by He-ion bombardment^9,27^, for co-circularly and cross-circularly polarized excitation and detection. We do not detect a valley polarization, as expected for a transition that occurs predominantly between localized defect levels.

## Discussion

We note that the absence of valley polarization for defect emission in monolayer transition metal dichalcogenides is non-trivial. For example, Hsu et al. reported a valley polarization for defect localized excitons in CVD-grown WSe_2_^45^. Similarly, Moody et al. reported valley polarization of defect localized excitons in exfoliated, electron beam irradiated WSe_2_^42^, which they assigned to defect-to-valence-band transitions. In both works, the defect emission was rather broad (about 60–100 meV) and the samples were neither in-situ prepared or encapsulated, such that adsorbates may have contributed to the observed defect emission. For clean and low-defect density WSe_2_, excitonic satellite peaks were recently demonstrated and assigned to bound-excitons complexes localized by residual unintentional dopants. In the latter study, a valley polarization of the satellite peak was observed as well^46^. In our case, we conclude that the X_L_ peak observed in our thermally annealed as well as He-ion treated MoS_2_ monolayers arises, due to a localized excitonic transition between the defect orbitals of the pristine sulfur vacancy.

Typically, the dominant emission process should involve the lowest energy state of the system, i.e., transitions of type D_1_, which according to the theoretical results may allow accessing defect-related valley polarizations in the Brillouin zone via their valley contributions near K and K′ (see also Supplementary Note 8). However, in our experiments, the defect emission is governed by transitions of intermediate energy, i.e., transitions of type D_2_, although the calculated oscillator strength varies only weakly in the relevant regime. A naïve scenario to explain our observations involves a relaxation cascade after the absorption process: optical excitation creates a free exciton, which gets localized, and then both hole and electron decay into a defect state (type D_2_). If further relaxation of the captured exciton into an excitonic state of type D_1_ is slow or prohibited, the emission will occur dominantly from the fully localized electron and hole state. From a theory point of view, the above picture demands not only going from absorption to emission, but also to include further interactions, such as exciton–exciton or exciton–phonon coupling^47^. The latter is motivated by the fact that previous work described the asymmetric line shape of defect emission by an independent boson model with a phonon bath^27^. Furthermore, in a recent study^30^, we found signatures of phonon replica corresponding to Raman shift of 248 cm^−1^ suggesting that the defect emission can couple also to localized optical phonon modes. Overall, these insights can provide starting points for the theoretical description of emission properties of such atomistic defects in highly confined two-dimensional material systems.

In summary, by combining far-field optical spectroscopy, atomic-resolution scanning probe microscopy, and ab initio theory, our study provides compelling evidence of optical defect emission from pristine sulfur vacancies in single layer MoS_2_. In contrast to previous studies, these pristine sulfur vacancies are generated in-vacuo or capped by hBN, and therefore, neither passivated by oxygen nor decorated with adsorbates. Similar to previous reports, we observe a broad L-band luminescence due to adsorbates in as-prepared MoS_2_ monolayers, which can be suppressed by a combination of h-BN encapsulation and mild annealing. In as-prepared layers and after mild annealing, pristine sulfur vacancies are absent, and oxygen passivated vacancies are the dominant defect. We suggest that oxygen-passivated vacancies form active sites for adsorption of molecular species, since many previous studies established a positive correlation between sulfur deficiency and defect emission^4,5,15^. Pristine vacancies are created in h-BN/MoS_2_/h-BN heterostructures either via in-vacuo thermal annealing or ex-situ helium ion bombardment, whereby the latter allows generating single photon emitters on demand^27^ with a position accuracy below 10 nm^9^. Guided by ab initio calculations, we identify transitions between a localized in-gap defect state and a localized resonant defect state as the most likely candidate.

## Methods

### Sample preparation

We micromechanically exfoliated synthetic MoS_2_ and hBN (NIMS, K.W. and T.T.) using adhesive tape. We used an all-dry viscoelastic stamping technique to transfer single flakes to a substrate consisting of SiO_2_/Si for PL characterization or epitaxial graphene on (6H)-SiC for STM/AFM characterization using a polydimethylsiloxane (PDMS) stamp. During the transfer, we heated the samples to approximately 60 °C, to increase the transfer probability. We cleaned the samples with acetone, isopropanol, and nitrogen gas after each stacking step to remove residues and increase the adhesion.

### In-vacuo annealing

For PL characterization, we annealed the MoS_2_/hBN/SiO_2_ samples in a modified optical cryostat (Janis ST-500). A customized heater was added to the cryostat for rapid thermal cycling between cryogenic temperatures (*T*_sample_ ∼20 K) and high temperatures (*T*_sample_ ∼900 K). We determined the annealing temperature at the sample with a thermocouple. We rapidly ramped to the desired annealing temperature and kept it constant for 30 min, then we cooled the sample with the highest possible rate back to cryogenic temperatures and conducted the PL measurements.

### Photoluminescence spectroscopy

The annealed samples (Figs. 1a–c,  2, and 4c) were studied with a custom microscope set-up (*λ*_excitation_ = 532 nm, Nikon Plan Fluor ELWD 20×/0.45, WD 7.4 mm). The cryostat was mounted on a motorized xy-stage (ASI) with a minimum stepsize of 100 nm. The emitted light was focused onto the entrance slit of the spectrometer (Andor Kymera 328, grating 300 grooves/mm), and the signal was collected by a CCD camera (Andor iXon). The photoluminescence of the ion-treated samples (Figs. 1d and  4c, d) was measured in a He-flow cryostat at *T* = 4.2–10 K (*λ*_excitation_ = 590 nm, Mitutoyo 100x/0.5 M Plan Apo NIR, WD 12 mm, PI Acton SP-2500i spectrometer, grating 300 grooves/mm). For valley polarization measurements, the circular polarization was adjusted using an achromatic λ/4-plate in front of the objective lens. The emitted light passed again through the same waveplate, and the polarization was analyzed using a linear polarizer.

### Scanning probe microscopy

We performed combined scanning tunneling microscopy and atomic force microscopy (Createc) at low temperatures (*T* ∼ 6 K) in vacuum with a base pressure of around 10^−10^ mbar. For STM/AFM, the MoS_2_/graphene/SiC samples were treated by mild annealing (*T* ∼ 500 K) under UHV conditions for 10–20 min. All STM images were recorded using the constant-current feedback and current setpoint of *I*_t_ = 100 pA. Chemically etched tungsten tips were sharpened by repeated indentations into a Au(111) substrate. All AFM images were recorded with a qPlus quartz crystal tuning fork in constant height mode with an applied bias voltage *V*_bias_ = 0 V.

### Helium ion microscopy

Single layer MoS_2_ flakes supported by graphene/SiC were nanostructured using a helium ion microscope (HIM ORION NanoFab, Zeiss). The whole MoS_2_ flake was exposed to a constant helium ion dose of 5 × 10^14^ cm^−2^. We operated the HIM at a helium pressure of 2.5 × 10^−6^ Torr, a beam energy of 30 keV, beam current of 0.7 pA, pixel spacing of 5 nm, and field of view (FOV) of 100 µm. To obtain the desired constant helium ion dose, the dwell time was adapted to the beam current.

### Ex-situ annealing and Raman spectroscopy

For ex-situ annealing studies, samples were annealed in a rapid thermal annealer under nitrogen atmosphere at a pressure of several mbar for 30–60 min at varying temperatures from 400 to 950 K. The cooling and heating ramp rate were about 10 K/s. After the annealing, the samples were taken out, exposed to ambient, and transferred into a vacuum chamber (base pressure < 10^−5^ mbar) for optical characterization. For Raman spectroscopy at room temperature, we used a WiTec Alpha 300 microscope (Zeiss Neofluar 60×, 0.6 NA with correction collar, 532 nm excitation wavelength, *P*_laser_ = 0.3 mW).

### Theoretical calculations

DFT calculations of the defect orbitals in Fig. 3 were performed using the Vienna Ab initio Simulation Package, VASP 5.4.4^48^. Details can be found in Supplementary Note 7. The GW-BSE calculations of the excitonic spectra of MoS_2_ with sulfur vacancies, shown in Fig. 4, were performed using the Quantum Espresso^49^ and the BerkeleyGW^50^ packages. The computational details are described in Supplementary Note 9.

## Supplementary information

Supplementary Information

## Data Availability

The datasets generated during and/or analysed during the current study are available from the corresponding authors on reasonable request.

## References

[1] Lin Z (2016). Defect engineering of two-dimensional transition metal dichalcogenides. 2D Mater..

[2] Chakraborty C, Vamivakas N, Englund D (2019). Advances in quantum light emission from 2D materials. Nanophotonics.

[3] Barthelmi K (2020). Atomistic defects as single-photon emitters in atomically thin MoS_2_. Appl. Phys. Lett..

[4] Carozo V (2017). Optical identification of sulfur vacancies: bound excitons at the edges of monolayer tungsten disulfide. Sci. Adv..

[5] Tongay S (2013). Defects activated photoluminescence in two-dimensional semiconductors: interplay between bound, charged and free excitons. Sci. Rep..

[6] Nan H (2014). Strong photoluminescence enhancement of MoS_2_ through defect engineering and oxygen bonding. ACS Nano.

[7] Greben K, Arora S, Harats MG, Bolotin KI (2020). Intrinsic and extrinsic defect-related excitons in TMDCs. Nano Lett..

[8] Korn T, Heydrich S, Hirmer M, Schmutzler J, Schüller C (2011). Low-temperature photocarrier dynamics in monolayer MoS2. Appl. Phys. Lett..

[9] Mitterreiter E (2020). Atomistic positioning of defects in helium ion treated single-layer MoS_2_. Nano Lett..

[10] Klingshirn, C. F. *Semiconductor Optics* (Springer, 2005).

[11] Wang G (2018). Colloquium: excitons in atomically thin transition metal dichalcogenides. Rev. Mod. Phys..

[12] Refaely-Abramson S, Qiu DY, Louie SG, Neaton JB (2018). Defect-induced modification of low-lying excitons and valley selectivity in monolayer transition metal dichalcogenides. Phys. Rev. Lett..

[13] Bretscher H (2021). Rational passivation of sulfur vacancy defects in two-dimensional transition metal dichalcogenides. ACS Nano.

[14] Chow PK (2015). Defect-induced photoluminescence in monolayer semiconducting transition metal dichalcogenides. ACS Nano.

[15] Kastl C (2019). Effects of defects on band structure and excitons in WS2 revealed by nanoscale photoemission spectroscopy. ACS Nano.

[16] Rosenberger MR, Chuang H-J, McCreary KM, Li CH, Jonker BT (2018). Electrical characterization of discrete defects and impact of defect density on photoluminescence in monolayer WS2. ACS Nano.

[17] Wu K (2018). Controllable defects implantation in MoS2 grown by chemical vapor deposition for photoluminescence enhancement. Nano Res..

[18] Edelberg D (2019). Approaching the intrinsic limit in transition metal diselenides via point defect control. Nano Lett..

[19] Gogoi PK (2017). Oxygen passivation mediated tunability of trion and excitons in MoS_2_. Phys. Rev. Lett..

[20] Lu J (2015). Bandgap engineering of phosphorene by laser oxidation toward functional 2D materials. ACS Nano.

[21] Sivaram SV (2019). Spatially selective enhancement of photoluminescence in MoS_2_ by exciton-mediated adsorption and defect Passivation. ACS Appl. Mater. Interfaces.

[22] Barja S (2019). Identifying substitutional oxygen as a prolific point defect in monolayer transition metal dichalcogenides. Nat. Commun..

[23] Schuler B (2019). Large spin-orbit splitting of deep in-gap defect states of engineered sulfur vacancies in monolayer WS2. Phys. Rev. Lett..

[24] Rogers C, Gray D, Bogdanowicz N, Mabuchi H (2018). Laser annealing for radiatively broadened MoSe2 grown by chemical vapor deposition. Phys. Rev. Mater..

[25] Venanzi T (2019). Exciton localization in MoSe2 monolayers induced by adsorbed gas molecules. Appl. Phys. Lett..

[26] Cadiz F (2017). Excitonic linewidth approaching the homogeneous limit in MoS_2_-based van der Waals heterostructures. Phys. Rev. X.

[27] Klein J (2019). Site-selectively generated photon emitters in monolayer MoS_2_ via local helium ion irradiation. Nat. Commun..

[28] Cho Y, Berkelbach TC (2018). Environmentally sensitive theory of electronic and optical transitions in atomically thin semiconductors. Phys. Rev. B.

[29] Hötger A (2021). Gate-switchable arrays of quantum light emitters in contacted monolayer MoS2 van der Waals heterodevices. Nano Lett..

[30] Klein, J. et al. Engineering the luminescence and generation of individual defect emitters in atomically thin MoS_2_. *ACS Photonics* **8**, 669–677 (2021).

[31] Tongay S (2013). Broad-range modulation of light emission in two-dimensional semiconductors by molecular physisorption gating. Nano Lett..

[32] Mouri S, Miyauchi Y, Matsuda K (2013). Tunable photoluminescence of monolayer MoS2 via chemical doping. Nano Lett..

[33] Li H, Huang M, Cao G (2016). Markedly different adsorption behaviors of gas molecules on defective monolayer MoS2: a first-principles study. Phys. Chem. Chem. Phys..

[34] Miremadi BK, Morrison SR (1986). Exfoliated MoS2 Temperature programmed desorption. Surf. Sci..

[35] Noh J-Y, Kim H, Kim Y-S (2014). Stability and electronic structures of native defects in single-layer MoS2. Phys. Rev. B.

[36] Komsa H-P, Krasheninnikov AV (2015). Native defects in bulk and monolayer MoS2 from first principles. Phys. Rev. B.

[37] Mignuzzi S (2015). Effect of disorder on Raman scattering of single-layer MoS_2_. Phys. Rev. B.

[38] Mitterreiter E (2019). In-situ visualization of hydrogen evolution sites on helium ion treated molybdenum dichalcogenides under reaction conditions. Npj 2D Mater. Appl..

[39] Lorchat E (2020). Filtering the photoluminescence spectra of atomically thin semiconductors with graphene. Nat. Nanotechnol..

[40] Amani M (2016). Recombination kinetics and effects of superacid treatment in sulfur- and selenium-based transition metal dichalcogenides. Nano Lett..

[41] McDonnell S, Addou R, Buie C, Wallace RM, Hinkle CL (2014). Defect-dominated doping and contact resistance in MoS_2_. ACS Nano.

[42] Moody G (2018). Microsecond valley lifetime of defect-bound excitons in monolayer WSe2. Phys. Rev. Lett..

[43] Rohlfing M, Louie SG (2000). Electron-hole excitations and optical spectra from first principles. Phys. Rev. B.

[44] Hybertsen MS, Louie SG (1986). Electron correlation in semiconductors and insulators: band gaps and quasiparticle energies. Phys. Rev. B.

[45] Hsu W-T (2015). Optically initialized robust valley-polarized holes in monolayer WSe_2_. Nat. Commun..

[46] Rivera P (2021). Intrinsic donor-bound excitons in ultraclean monolayer semiconductors. Nat. Commun..

[47] Chen H-Y, Sangalli D, Bernardi M (2020). Exciton-phonon interaction and relaxation times from first principles. Phys. Rev. Lett..

[48] Kresse G, Furthmüller J (1996). Efficiency of ab-initio total energy calculations for metals and semiconductors using a plane-wave basis set. Comput. Mater. Sci..

[49] Giannozzi P (2009). QUANTUM ESPRESSO: a modular and open-source software project for quantum simulations of materials. J. Phys. Condens. Matter.

[50] Deslippe J (2012). BerkeleyGW: a massively parallel computer package for the calculation of the quasiparticle and optical properties of materials and nanostructures. Comput. Phys. Commun..

