# Supplementary material for: The role of chalcogen vacancies for atomic defect emission in MoS2
Source: Nat Commun. 2021 Jun 22;12:3822. doi: 10.1038/s41467-021-24102-y (PMC8219741; doi:10.1038/s41467-021-24102-y)
Supplement: Supplementary file 1 — Supplementary Information [file 41467_2021_24102_MOESM1_ESM.pdf]

Supplementary Information for

The role of chalcogen vacancies for atomic defect emission in MoS_2

Elmar Mitterreiter[1,2], Bruno Schuler[3,4], Ana Micevic[1,2], Daniel Hernangómez-Pérez[5], Katja Barthelmi[1,2], Katherine A. Cochrane[3], Jonas Kiemle[1,2], Florian Sigger[1,2], Julian Klein[1,6], Edward Wong[3], Edward S. Barnard[3], Kenji Watanabe[7], Takashi Taniguchi[8], Michael Lorke[9,10], Frank Jahnke[10], Johnathan J. Finley[1,2], Adam M. Schwartzberg[3], Diana Y. Qiu[11], Sivan Refaely-Abramson[5], Alexander W. Holleitner[1,2*], Alexander Weber-Bargioni[3*], Christoph Kastl[1,2*]

1 – Walter Schottky Institut and Physics Department, Technical University of Munich, Am Coulombwall 4a, 85748 Garching, Germany

2 – Munich Center for Quantum Science and Technology (MCQST), Schellingstrasse 4, 80799 München, Germany

3 – Molecular Foundry, Lawrence Berkeley National Laboratory, 1 Cyclotron Road, Berkeley, CA 94720, United States

4 – nanotech@surfaces Laboratory, Empa – Swiss Federal Laboratories for Materials Science and Technology, 8600 Dübendorf, Switzerland

5 – Department of Molecular Chemistry and Materials Science, Weizmann Institute of Science, Rehovot, Israel

6 – Department of Materials Science and Engineering, Massachusetts Institute of Technology, Cambridge, MA 02139, United States

7 – Research Center for Functional Materials, National Institute for Materials Science, 1-1 Namiki, Tsukuba 305-0044, Japan

8 – International Center for Materials Nanoarchitectonics, National Institute for Materials Science, 1-1 Namiki, Tsukuba 305-0044, Japan

9 – Bremen Center for Computational Materials Science, University of Bremen, Am Fallturm 1, 28359 Bremen, Germany1

10 – Bremen Institute for Theoretical Physics, University of Bremen, Otto-Hahn-Allee 1, 28359 Bremen, Germany

11 – Department of Mechanical Engineering and Materials Science, Yale University, New Haven, CT, USA

*holleitner@wsi.tum.de, afweber-bargioni@lbl.gov, christoph.kastl@wsi.tum.de

Supplementary Note 1 - Details of in-vacuo annealing procedure

The details of the custom-built in-vacuo annealing setup together with a representative annealing and cooling cycle are depicted in Supplementary Figure 1. Our custom-built setup allows to cycle between high-temperature annealing steps and cryogenic photoluminescence measurements without breaking the vacuum, i.e. without exposure of the sample to ambient conditions.

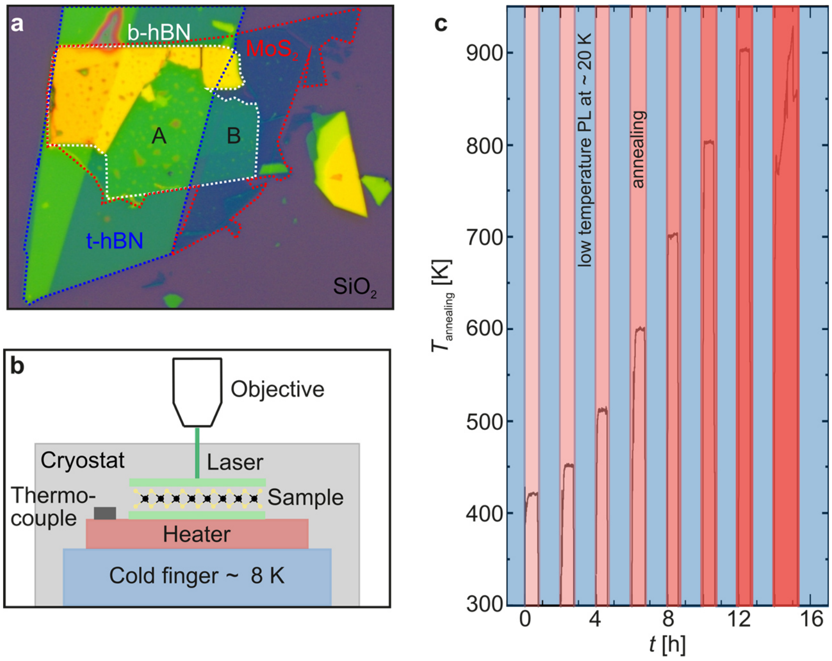

Supplementary Figure 1: Schematic of in-vacuo annealing. a Microscope image of a sample consisting of bottom hBN (b-hBN), MoS_2, and top hBN (t-hBN) on SiO_2. A and B represent the resulting fully, and half-encapsulated MoS_2 heterostructure, respectively. **b** Schematic measurement setup showing a modified optical cryostat with integrated heater enabling in-vacuo high-temperature annealing (up to $T_{\text{annealing}} \sim 900$ K) and successive low-temperature PL measurements ($T_{\text{sample}} \sim 20$ K). **c** Temperature profile of the entire PL study over time (t). The red-shaded areas highlight the temperature profile during the annealing processes. Upon reaching the desired annealing temperature $T_{\text{annealing}}$, the latter was maintained constant for 30 minutes. The blue-shaded areas represent the low-temperature PL measurements.

Supplementary Note 2 - Extended dataset of annealing induced defect luminescence

Supplementary Figure 2 depicts an extended dataset of the photoluminescence of single layer MoS2 after different annealing steps, both for fully encapsulated (top and bottom hBN) as well as for half encapsulated MoS2 (bottom hBN only). At the largest annealing temperatures material degradation is observed.

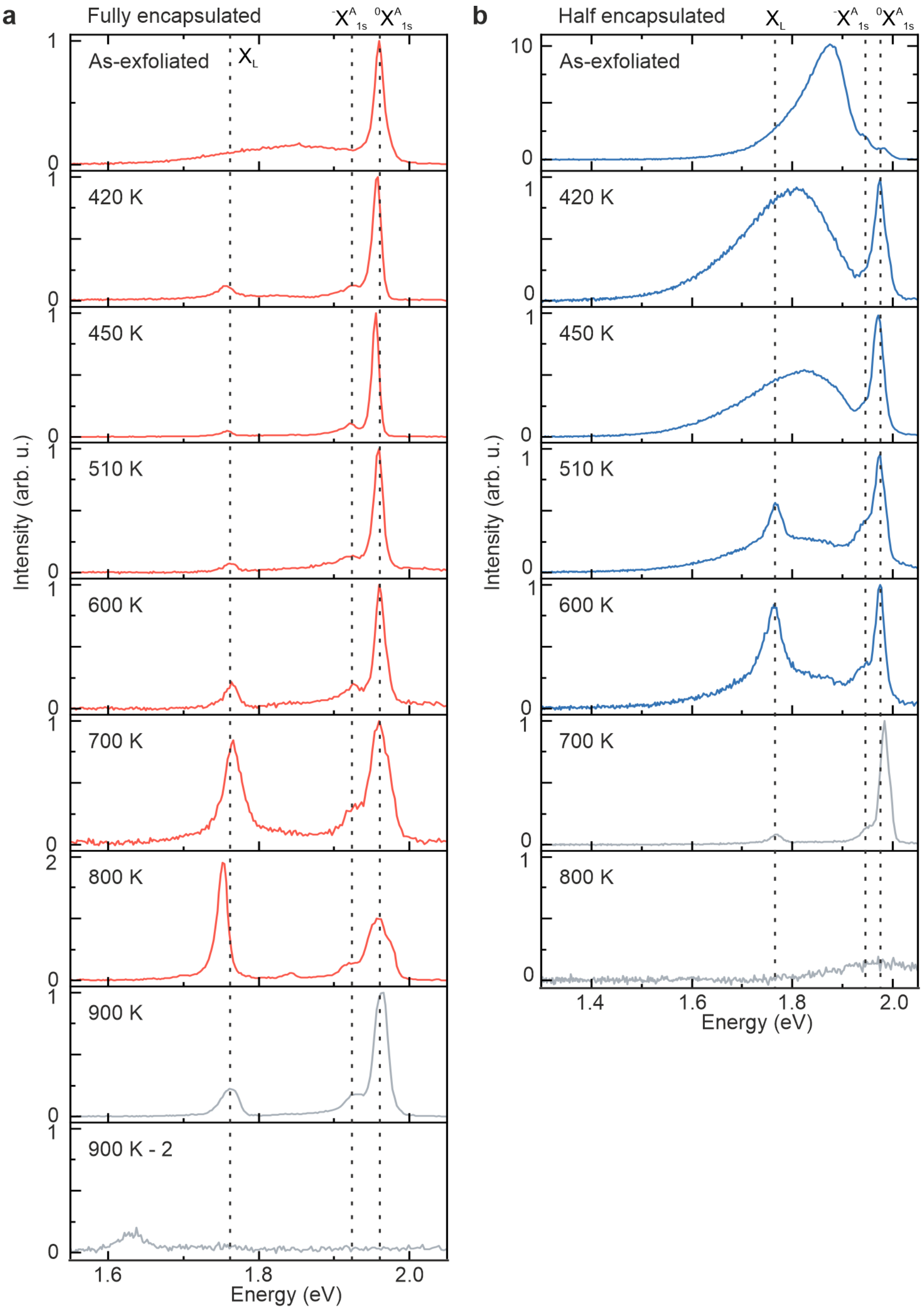

Supplementary Figure 2: Annealing temperature dependent evolution of low-temperature photoluminescence (PL) spectra. a PL spectra of fully encapsulated pristine MoS_2, and after annealing to 420 K, 450 K, 510 K, 600 K, 700 K, 800 K, 900 K, and again 900 K. The dashed lines indicated the position of the neutral exciton (^0X_A), the trion (^-X_A), and the defect luminescence (X_L). Pristine MoS_2 exhibits a broad emission peak, which is strongly

reduced after mild annealing. A single emission peak appears, and its intensity increases with rising annealing temperature. The intensity of the single emission peak decreases after annealing to 900 K and the PL disappears after a subsequent annealing step to 900 K (gray lines). **b** PL spectra of pristine MoS_2 on bottom h-BN only (no top hBN), and after annealing to 420 K, 450 K, 510 K, 600 K, 700 K, and 800 K. A similar behavior to fully encapsulated MoS_2 occurs. However, lower annealing temperatures of about 700 K already reduce the intensity of single emission line before it vanishes at 800 K (gray lines).

Supplementary Note 3 - Power dependence of defect and exciton luminescence

Supplementary Figure 3 shows the laser power dependence of the low temperature photoluminescence of single layer MoS_2 with annealing induced defects. The defect emission shows a clear saturation behavior as function of laser excitation power, which is consistent with a finite number of defect states. The neutral exciton emission shows a non-saturating, linear behavior as function of laser excitation power.

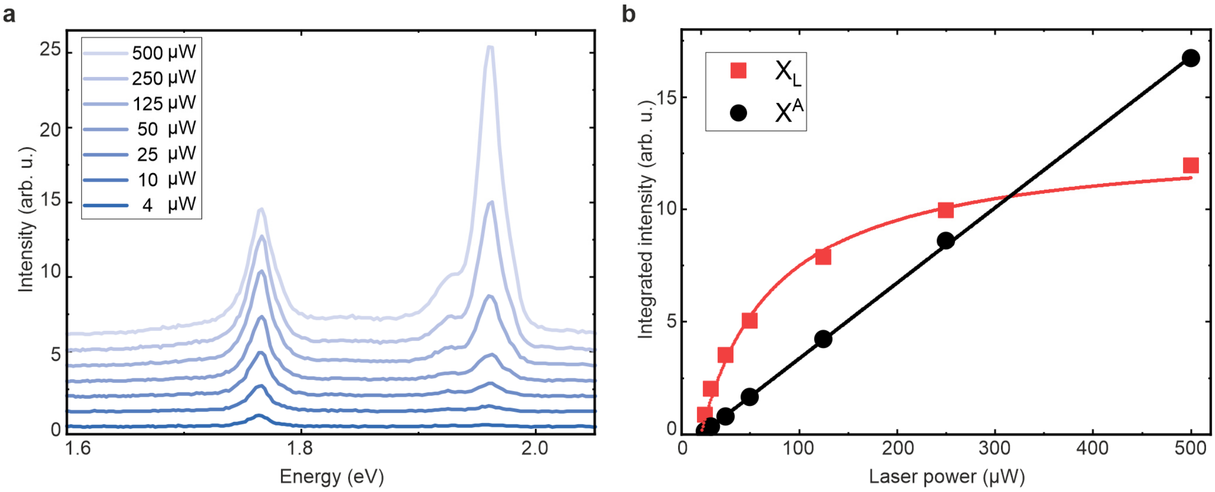

Supplementary Figure 3: Laser power dependent PL of fully encapsulated MoS_2 after annealing to 800 K. a Power dependent PL spectra. Curves are offset for clarity. **b** Power dependent integrated intensity of the neutral exciton (X_A) (red) and the single emission line X_L (black). We do not observe a laser power dependent saturation for the emission of the exciton, i.e. the power law fit is linear (black line). However, the defect emission line exhibits a saturating behavior, which we can be fitted by $I \propto \frac{P}{P + P_{Sat}}$ (red line). The turnover for saturation where $P = P_{sat}$ occurs at approximately 50 μW.

Supplementary Note 4 – Estimation of absolute defect density

Supplementary Figure 4 depicts the results of an ex situ annealing and Raman study, which was used to estimate the absolute value of the thermally induced defect density. The defect density was estimated from the defect-induced shift of the characteristic Raman modes based on a phonon confinement model.

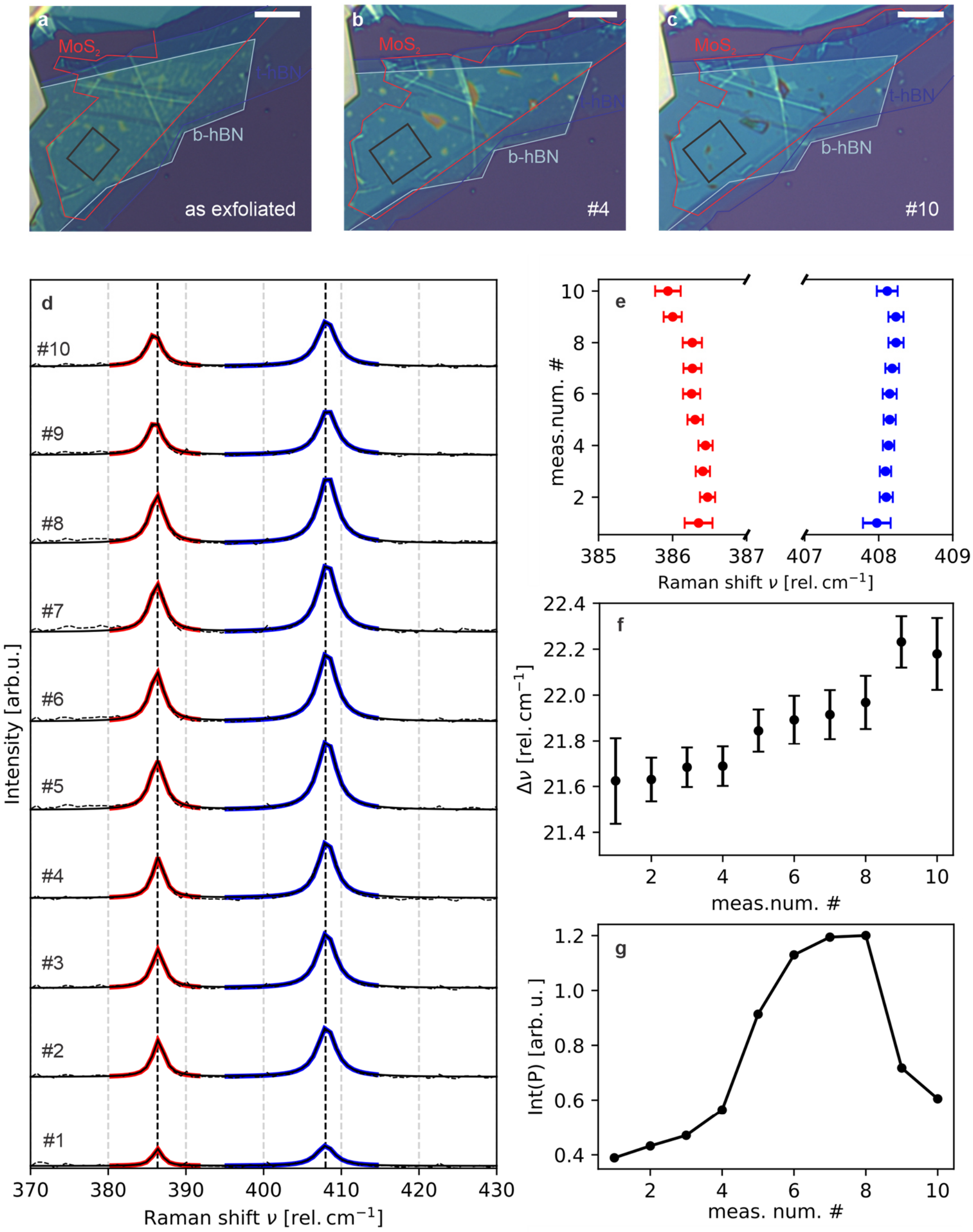

h

meas.num #	1	2	3	4	5	6	7	8	9	10
T [K]	510	600	650	700	750	750	750	850	900	950
t [s]	1800	1800	1800	1800	1800	1800	3600	1800	1800	1800
P_D [10^{14}cm^{-2}]	0.057	0.061	0.094	0.097	0.194	0.223	0.238	0.27	0.434	0.402

Supplementary Figure 4: Ex-situ annealing and Raman study. Optical microscope images of a monolayer MoS_2 (red outline) encapsulated in top (t-hBN, dark blue outline) and bottom hBN (b-hBN, light blue outline) for different stages of the annealing study. **a** In the as-exfoliated sample trapped polymer residues and bubbles are clearly visible. **b** After the forth annealing step (700 K), the polymer residues have aggregated to larger clusters leaving large clean areas. **c** After the tenth annealing step, the sample is virtually free from polymer residues. **d** Evolution of the room temperature Raman spectra after 10 consecutive annealing steps (#1 - #10). Each spectrum was averaged over the area on the sample indicated by the black rectangle in (a). The colored lines denote Lorentzian fits to the peaks (red: E'-mode, blue: A'_{1g}-mode). The induced defects alter the Raman fingerprint of MoS_2 and allow the correlation between defect density and first-order Raman mode splitting according to a phonon confinement model [1,2]. **e** With increasing annealing temperature, the E'-mode (A'_{1g}-mode) shifts to lower (higher) wavenumbers. The error bars denote the fit error of the Raman peak centers from (d). **f** The mode splitting between the E'-mode A'_{1g}-mode ($\Delta\nu$) increases monotonously with annealing time and temperature within the experimental uncertainty. The error bars include the fit error of the Raman peak centers from (d). **g** Evolution of the room temperature integrated photoluminescence intensity, denoted Int(P), after 10 consecutive annealing steps (#1 - #10). Initially, the photoluminescence intensity increases upon annealing, which may be attributed to the increased optical quality of the heterostructure (cf. optical images) or to a beneficial effect of shallow defects on the overall PL quantum yield[3] or a combination of both. At very high annealing temperatures ($T \geq 900$ K), the PL intensity drops significantly indicating a deleterious effect of the high density of thermally induced defects. This finding is consistent with the data in Supplementary Figure 2, where sample degradation occurred at a nominal annealing temperature of 900 K for a fully encapsulated layer of MoS_2. **h** Table indicating the different annealing temperatures (T) and annealing times (t) corresponding to each step. Furthermore, we estimated the corresponding defect density (P_D) based on the defect-induced mode splitting. From our previous study on He-ion induced defects[4], we determined a close to linear correspondence between defect density and shift of the mode spacing. In these studies, the absolute number of the defect density was calculated based on the known He-ion dose and the sputtering yield calculated from Monte-Carlo simulations and independently corroborated from scanning probe measurements[5]. Within the limitations of this approach, we can now give a quantitative estimate of the absolute number of induced defects based on the ex-situ Raman spectroscopy. We note that for the defect density after the first annealing step (510 K), we assume the background density value determined from our previous scanning probe study[5].

Supplementary Note 5 – Absence of defect luminescence in MoS_2 supported on graphene

Supplementary Figure 5 shows the low temperature PL spectrum of single layer MoS2 supported on graphene and exposed to He-ion bombardment. The defect emission is absent due to fast quenching of the slow defect luminescence.

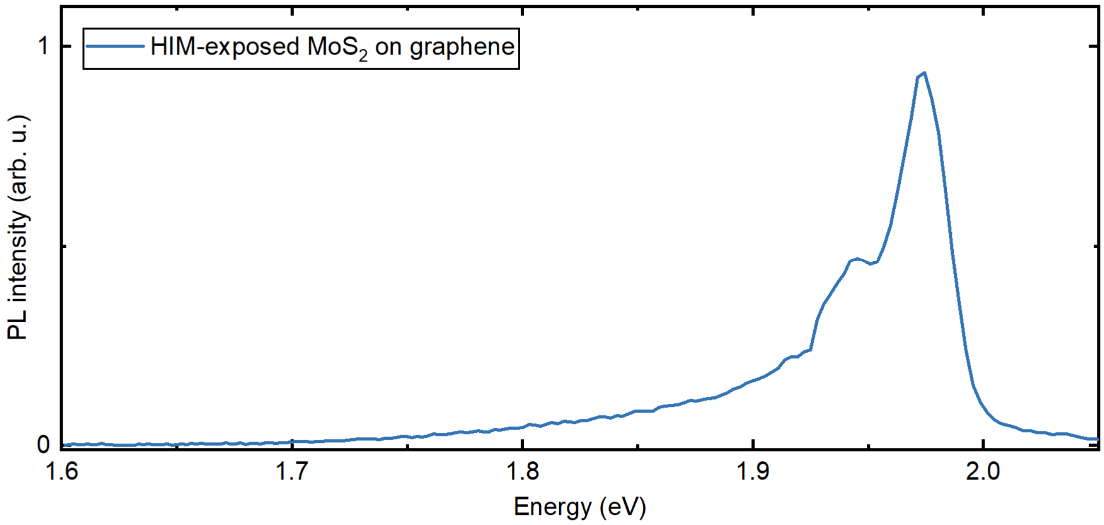

Supplementary Figure 5: Low-temperature PL spectrum of homogeneously He-ion (HIM) irradiated single-layer MoS_2 on graphene/SiC. The He-ion dose was $5 \cdot 10^{14}$ cm^{-2}. The defect emission is absent due to quenching from the underlying graphene substrate.

Supplementary Note 6 – Scanning tunneling microscopy of MoS_2 after mild annealing

Supplementary Figure 6 displays a representative scanning tunneling microscope image of single layer MoS_2 supported on graphene after only mild annealing steps ($T_{annealing}$ < 500 K). The surface appears virtually free from adsorbates. Within our experimental statistics, the dominant defects are oxygen atoms substituting for sulfur either in the top sulfur lattice (O_S top) or in the bottom sulfur lattice (O_S bottom).

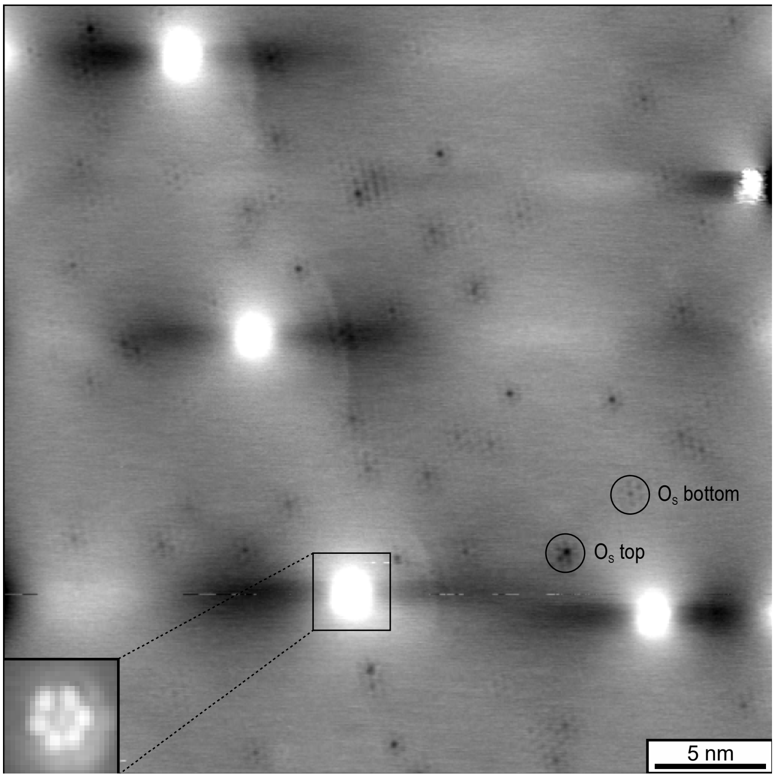

Supplementary Figure 6: Scanning tunneling microscope overview image of single layer MoS_2 on graphene/SiC after mild annealing. Within our experimental statistics, the only observed defects are oxygen passivated sulfur top and bottom vacancies (O_S top and O_S bottom). The bright spots correspond to adsorbed molecules on the MoS_2 surface.

Supplementary Note 7 - Details of DFT calculations for vacancy orbitals

DFT calculations were performed using the Vienna Ab initio Simulation Package, Vasp 5.4.4[6,7], using the projector augmented wave method and treating the semi-core d-states as part of the valence shell in the PBE variant of the generalized gradient approximation[8]. For defect calculations, 9×9 supercells, consisting of 243 atoms were used with the experimental lattice constant, applying the G-point approximation. The defect geometries were fully relaxed. A 450 eV (900 eV) cutoff was applied for the expansion of the wave functions (charge density).

Supplementary Note 8 – Calculations of k-resolved band contributions of the excitonic states in MoS_2 with sulfur vacancies

Supplementary Figure 7 provides k-resolved band contributions of the excitonic states in MoS_2 with sulfur vacancies. The neutral, free exciton (zero detuning) and the lowest defect transition (~ -0.6 eV detuning) have dominant contributions from the K-point, suggesting a valley polarization in the optical transition.

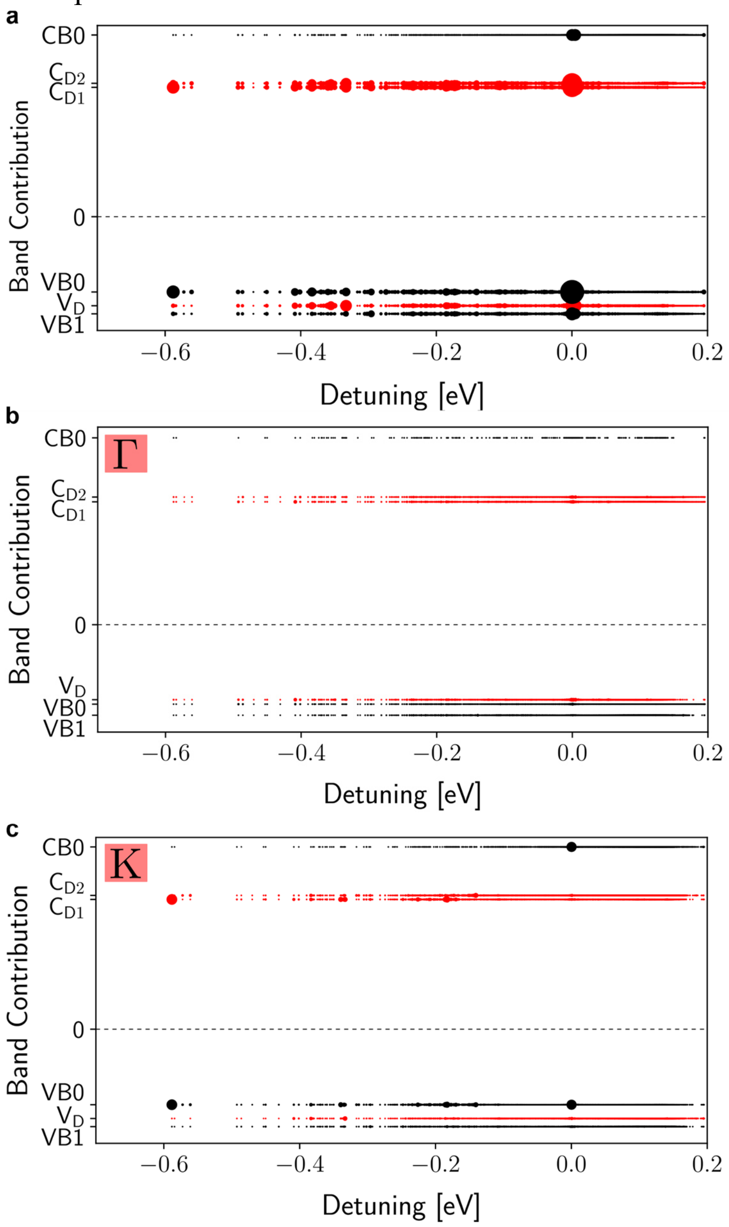

Supplementary Figure 7: k-resolved band contributions of the excitonic states in MoS_2 with sulfur vacancies. a Calculated excitonic spectrum of a monolayer of MoS_2 (in vacuum) with sulfur vacancies. The strong electron-interaction leads to a large manifold of excitonic states. Each set of dots at a given energy, referenced to the usual free A-exciton transition (zero detuning corresponds to an energy of 2.15 eV) describes an excitonic state. The size of the dots is proportional to the relative contributions from the different bands. For clarity, we only show the spin-up channel (single valley). Black indicates delocalized band states and red indicates localized defect states. CB0 and VB0, VB1 denote the dispersive conduction and valence bands

(above and below the Fermi level), respectively. C_{D1} and C_{D2} are localized defect levels, split by spin-orbit interaction arising from conduction band states. V_D is the localized defect level arising from the valence band. **b** Band contributions evaluated at the Γ-point (the size of each dot has been multiplied by a factor of 10 for clarity). Note the different energetic order of the valence states due to the band dispersion. **c** Band contributions from the K-point (the size of each dot has been multiplied by a factor of 10 for clarity).

Supplementary Note 9 - Details of GW-BSE calculations for absorbance spectra of MoS_2 with sulfur vacancies

We first performed density functional theory (DFT) calculations within the local density approximation (LDA) using the Quantum-ESPRESSO code[9]. The calculations were done on a 5×5 supercell arrangement of a MoS_2 monolayer with one S vacancy, following prior work[10]. We used a plane-wave basis and norm-conserving pseudopotentials with a 85 Ry plane-wave cutoff. We included the Mo semi-core 4d, 4p, 4s, 5s and 5p states as valence states in our pseudopotential. The distance between repeated supercells in the out-of-plane direction was 15 Å. We relaxed the geometry within DFT and used the DFT charge density (converged in a 6×6×1 k-grid) as a starting point for our GW and Bethe Salpeter equation (GW-BSE) calculations. Our GW calculations are performed with the BerkeleyGW code[11] using the generalized plasmon-pole model[12], an energy cutoff of 25 Ry for the plane-wave components of the dielectric matrix, and a nonuniform k-point sampling corresponding to a 30×30 uniform k-grid[13]. We included about 2,200 unoccupied states in the sum over empty states in the self-energy and polarizability. To compute the exciton transitions, we shifted the DFT energy levels to the computed GW values at the Γ-point, and coupled electron and hole states through the BSE formalism within the BerkeleyGW code[11,14]. We diagonalized the BSE Hamiltonian within the Tamm-Dancoff approximation with 3 valence and 3 conduction bands, and we interpolated to a uniform k-grid of 18×18×1 k-points. A 6x6x1 k-point grid and a 5 Ry cutoff were used for the plane-wave components of the dielectric matrix used in the BSE. Spin orbit coupling was added as a perturbation as in Ref. [10]. We find that these parameters converge the calculated exciton excitation energies up to 200 meV.

Supplementary References

1. Klein, J. *et al.* Robust valley polarization of helium ion modified atomically thin MoS_2. *2D Mater.* **5**, 011007 (2017).
2. Mignuzzi, S. *et al.* Effect of disorder on Raman scattering of single-layer Mo S 2. *Phys. Rev. B* **91**, 195411 (2015).
3. Goodman, A. J., Willard, A. P. & Tisdale, W. A. Exciton trapping is responsible for the long apparent lifetime in acid-treated MoS_2. *Phys. Rev. B* **96**, 121404 (2017).
4. Mitterreiter, E. *et al.* In-situ visualization of hydrogen evolution sites on helium ion treated molybdenum dichalcogenides under reaction conditions. *Npj 2D Mater. Appl.* **3**, 25 (2019).
5. Mitterreiter, E. *et al.* Atomistic Positioning of Defects in Helium Ion Treated Single-Layer MoS $_2$. *Nano Lett.* **20**, 4437–4444 (2020).
6. Kresse, G. & Furthmüller, J. Efficiency of ab-initio total energy calculations for metals and semiconductors using a plane-wave basis set. *Comput. Mater. Sci.* **6**, 15–50 (1996).
7. Kresse, G. & Furthmüller, J. Efficient iterative schemes for ab initio total-energy calculations using a plane-wave basis set. *Phys. Rev. B* **54**, 11169–11186 (1996).
8. Perdew, J. P., Burke, K. & Ernzerhof, M. Generalized Gradient Approximation Made Simple. *Phys. Rev. Lett.* **78**, 1396–1396 (1997).
9. Giannozzi, P. *et al.* QUANTUM ESPRESSO: a modular and open-source software project for quantum simulations of materials. *J. Phys. Condens. Matter* **21**, 395502 (2009).
10. Refaely-Abramson, S., Qiu, D. Y., Louie, S. G. & Neaton, J. B. Defect-Induced Modification of Low-Lying Excitons and Valley Selectivity in Monolayer Transition Metal Dichalcogenides. *Phys. Rev. Lett.* **121**, 167402 (2018).
11. Deslippe, J. *et al.* BerkeleyGW: A massively parallel computer package for the calculation of the quasiparticle and optical properties of materials and nanostructures. *Comput. Phys. Commun.* **183**, 1269–1289 (2012).
12. Hybertsen, M. S. & Louie, S. G. Electron correlation in semiconductors and insulators: Band gaps and quasiparticle energies. *Phys. Rev. B* **34**, 5390–5413 (1986).
13. da Jornada, F. H., Qiu, D. Y. & Louie, S. G. Nonuniform sampling schemes of the Brillouin zone for many-electron perturbation-theory calculations in reduced dimensionality. *Phys. Rev. B* **95**, 035109 (2017).
14. Rohlfing, M. & Louie, S. G. Electron-Hole Excitations in Semiconductors and Insulators. *Phys. Rev. Lett.* **81**, 2312–2315 (1998).
